# Impact of Dietary Protein Content on Soil Bacterial and Fungal Communities in a Rice–Crab Co-culture System

**DOI:** 10.3389/fmicb.2021.696427

**Published:** 2021-06-21

**Authors:** Yingdong Li, Lisong Li, Yilin Yu, Qingbiao Hu, Xiaodong Li

**Affiliations:** Key Laboratory of Livestock Infectious Diseases in Northeast China, Ministry of Education, College of Animal Science and Veterinary Medicine, Shenyang Agricultural University, Shenyang, China

**Keywords:** MiSeq, rice crab coculture system, soil bacterial community, dietary protein content, soil fungal community, soil community structure, nutrient translocation, alpha diversity

## Abstract

Although co-culture of paddy fields with aquatic animals is lucrative, the ecological impacts of high-protein content entering the agricultural soil via animal pellet feed and feces have not been well studied. Moreover, the effects of dietary protein on soils and soil microbes remain unclear. To elucidate this, we examined soil bacterial and fungal community composition and temporal changes in paddy fields subjected to different protein-content diets via 16S/18S rRNA gene amplicon sequencing analysis with a high-throughput next-generation sequencer. MiSeq sequencing revealed that protein content significantly impacted fungal community structure. High-protein diets reduced bacterial community diversity and relative abundance in both July and October. The phylum-level bacterial taxonomic composition was not affected by diet treatment, while in fungi, a major phylum-level shift was evident. Hierarchically clustered analysis showed that high-protein diets significantly reduced the relative abundance of *Brevundimonas* in both July and October. Saprotrophic macrofungal diversity was negatively related to dietary protein content. Considering microbial community structure and environmental factors, ca. 15% protein content is appropriate for the rice-crab co-culture system that we studied.

## Introduction

Rice is a staple food for over half of the global population, and its production has increased in the last two decades due to technological advances, including the use of pesticides and chemical fertilizers. Although improving grain yield and rice quality are important goals for many governments, minimizing chemical fertilizer and pesticide inputs is important for long-term agricultural and environmental sustainability (Yao et al., 2017; Stuart et al., 2018). Rice fields can provide a habitat mosaic of temporary and more permanent waters and sustain populations of various aquatic species, including carp, crabs, crayfish, soft-shelled turtles, and frogs (Lawler, 2001). Rice-aquatic-animal co-culture has been widely studied; such systems can contribute to the ecological intensification of agriculture by providing multiple ecosystem services, promoting biological pest control, reducing the use of pesticides, improving soil quality, and enhancing crop yields (Xie et al., 2011; Hu et al., 2016; Sha et al., 2017; Wan et al., 2019). Moreover, such systems increase protein production for human consumption and reduce water use and environmental pollution (Bashir et al., 2020). Thus, co-culture systems have gained increasing attention in recent years because of their benefits.

In China, the government has established many projects to help farmers develop diverse paddy field co-culture models, including rice-duck, rice-fish, rice-crayfish, and rice-crab. However, high-protein diets widely used in pond aquaculture are also used in rice-aquatic animal co-culture. Undigested protein-rich feed and feces are excreted into the bottom soil of paddy fields, potentially causing severe nitrogen pollution of the soil and irrigation water. However, studies have shown that rice-animal co-culture can enhance nutrient use efficiency and reduce nutrient loss to the environment via the complementary use of nutrients by aquatic animals and rice (Hu et al., 2020). Nonetheless, the relationships between high-protein diets and paddy field soil conditions in co-culture systems remain unclarified.

Rice-crab co-culture is the characteristic rice-based ecological aquaculture system used in northern China (Song et al., 2019; Wang et al., 2019). It improves soil nutrient levels and increases soil-to-rice nutrient translocation capacity (Bashir et al., 2021). Although high-throughput sequencing (e.g., Illumina) is increasingly used to analyze rice soil microbial diversity and community structure, the microbial response to different rice-crab co-culture systems is yet to be adequately studied. Determining soil bacterial community structure in rice-crab co-culture systems can provide key initiatives for improving aquaculture. Moreover, the soil bacterial community structure has a strong influence on the bacterial microbiota of crabs. The relationships between rice-crab co-culture soil microbiota communities and dietary protein content have not previously been studied. Therefore, we examined these dynamics and aimed to provide a theoretical reference for these relationships.

## Materials and Methods

### Ethics Statement

Our study did not involve endangered or protected species. In China, breeding and catching Chinese mitten crabs, *Eriocheir sinensis*, in rice fields does not require specific permits. All efforts were made to minimize animal suffering and discomfort. The experimental protocol was approved by the Animal Ethics Committee of Shenyang Agriculture University.

### Field Description and Experimental Design

The experiments were performed in 12 paddy fields (each 6 × 7 m^2^) at Panjin Guanghe Crab Industry Co., Ltd., Panjin, Liaoning Province, China, during the 2020 rice-growing season (over 5 months). In total, 30,000 late megalopa-stage Chinese mitten crabs (average weight was 6.25 ± 1.21 mg) were obtained from Panjin Guanghe Crab Industry and were randomly distributed in the 12 fields (2,500 crabs/field). Three different protein-content diets were formulated (15, 30, and 45% crude protein). As a control, no supplemental food was provided (Table 1). During the experiment, crabs were fed at 10% of body weight per day (at 08:00, 12:00, and 18:00) for 5 months.

**TABLE 1:** Composition and nutrient levels of the basal diet (as-feed basis, %).

Groups and sample time		Pellet content					Nutritional composition	
July 15th	October 1st	Fish meal (%)	Soybean meal (%)	Wheat meal (%)	Fish oil (%)	Others (%)	Crude protein (%)	Crude lipid (%)
JTD_0	OTD_0	–	–	–	–	–	–	–
JTD_15	OTD_15	6	3	72.59	7.5	10.91	15.01	9.13
JTD_30	OTD_30	27	13.5	43.09	5.5	10.91	30.49	9.27
JTD_45	OTD_45	47	23.5	15.09	3.5	10.91	45.21	9.31

### Sample Collection

Soil samples were collected on July 15, 2020, during the tillering stage (15 days after crab introduction), and October 1, during the ripening stage (100 days after seed sowing). For each plot, five soil samples (diameter 2.5 cm and depth 0–20 cm) were pooled to obtain one biological replicate. After removing rice roots and stones, the pooled soil sample was placed in a sterile plastic bag in an icebox until transport to a laboratory for further sample storage and pH analysis. pH was quantified using a PHS-3C pH meter (Shanghai, China) with 1:2.5 vol soil/H_2_O solutions.

### DNA Extraction and Purification

Soil samples were homogenized, and a 0.8-g subsample of soil was used for total genomic DNA extraction. DNA was extracted using the OMEGA Soil DNA kit (Omega Bio-Tek, Norcross, GA) according to the manufacturer’s instructions and stored at –20°C before further analysis. The quantity and quality of the extracted DNA were evaluated using a NanoDrop ND-1000 spectrophotometer (Thermo Fisher Scientific, Waltham, MA) and agarose gel electrophoresis, respectively.

### 16S rRNA Gene and ITS Amplicon Sequencing

The V5–V7 regions of the bacterial 16S ribosomal RNA genes were amplified by PCR using the forward primer 799 F (5′-AACMGGATTAGATACCCKG-3′) and reverse primer 1193R (5′-ACGTCATCCCCACCTTCC-3′). The forward primer ITSF (5′-CTTGGTCATTTAGAGGAAGTAA-3′) and reverse primer ITSR (5′-GCTGCGTTCTTCATCGATGC-3′) were used to amplify the fungal ITS regions.

Thermal cycling consisted of initial denaturation at 98°C for 5 min, followed by 28 cycles of denaturation at 98°C for 30 s, annealing at 55°C for 30 s, and extension at 72°C for 45 s, with a final extension of 5 min at 72°C. Amplicons were extracted from 2% agarose gels, purified using Vazyme VAHTS DNA Clean Beads (Vazyme, Nanjing, China) according to the manufacturer’s instructions, and quantified using the Quant-iT PicoGreen dsDNA Assay Kit (Invitrogen, Carlsbad, CA). The soil sample bacterial community diversity and composition were analyzed based on the raw sequencing data obtained using the Illumina MiSeq platform at Shanghai Personal Biotechnology Co., Ltd. (Shanghai, China), according to standard protocols.

### Bioinformatics and Statistical Analysis

After sequencing, raw FASTQ data were processed using QIIME2 and R v. 3.2.0, with slight modifications according to the official tutorials^1^. Briefly, the raw sequence data were demultiplexed, quality filtered, denoised, and merged, and the chimeras were removed using the DADA2 plugin. Amplicon sequencing variant (ASV)-level alpha diversity indices (Chao1 richness estimator, observed species, Shannon diversity index, Simpson index, Faith’s phylogenetic diversity index, Pielou’s evenness, and Good’s coverage) were calculated using the ASV table in QIIME2 and were visualized using box plots. Taxonomic composition and abundance were investigated using MEGAN and GraPhlAn, respectively. A Venn diagram was generated using the R package “VennDiagram” to visualize the shared and unique ASVs among samples or groups, based on the occurrence of ASVs across samples and groups, regardless of their relative abundance. The raw reads have been deposited with the NCBI (BioProject number PRJNA693650).

## Results

### Analysis of Pyrosequencing Data

In total, 55,522 bacterial and 75,786 fungal non-singleton sequences were obtained from all 24 samples (Supplementary Table 1), with average read lengths of 248 bp for bacterial sequences (ranging 78–431 bp) and 358 bp for fungal sequences (ranging 135–441 bp) (Supplementary Figures 1, 2). After sequence processing and quality filtering, 88,281 bacterial OTUs and 4,356 fungal OTUs were identified (Supplementary Figures 3, 4). The observed and estimated community structure indices were higher for bacterial than for fungal OTUs (Supplementary Table 2). The pH data of those soil samples are shown in Supplementary Table 3.

### Bacterial and Fungal Community Structure

**FIGURE 1:**
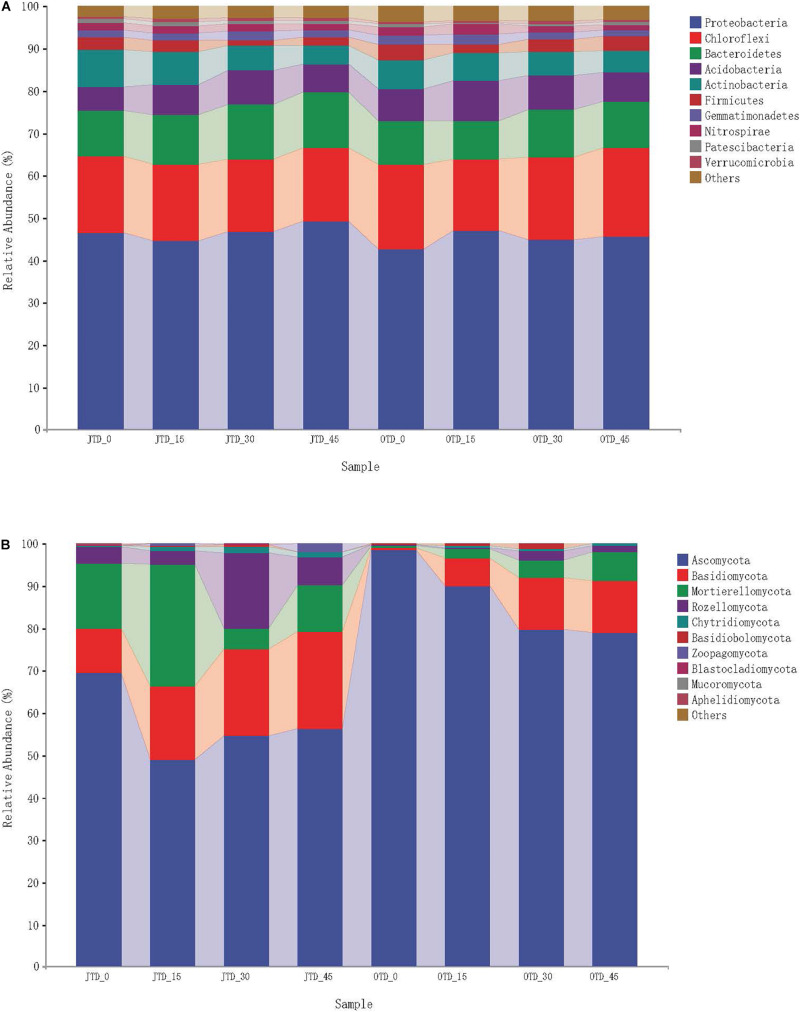
Relative abundance of bacterial group **(A)** and fugal group **(B)** under different protein content diets treatments in July and October.

The ten most abundant soil bacterial phyla were Proteobacteria, Chloroflexi, Bacteroidetes, Acidobacteria, Actinobacteria, Firmicutes, Gemmatimonadetes, Nitrospirae, Patescibacteria, and Verrucomicrobia (Supplementary Figure 5A). The phylum-level bacterial taxonomic composition was not affected by diet treatment, while in fungi, a major phylum level shift was evident (Figure 1A). Five bacterial phyla (Proteobacteria, Chloroflexi, Bacteroidetes, Acidobacteria, and Actinobacteria) accounted for 90% of the sequences.

Regarding the fungal communities, 10 phyla were observed in the soil samples: Ascomycota, Basidiomycota, Mortierellomycota, Rozellomycota, Chytridiomycota, Basidiobolomycota, Zoopagomycota, Blastocladiomycota, Mucoromycota, and Aphelidiomycota (Supplementary Figure 5B). Fungal community composition underwent major phylum-level shifts between July and October (Figure 1B): Ascomycota dominated in October (especially in the control group), and Basidiomycota comprised a large proportion of the fungal community in the OTD_15, _30, and _45 groups.

### Microbial Alpha and Beta Diversity

Table 2 shows the bacterial and fungal within-habitat (alpha) diversity. According to the Chao1 analysis. The bacterial richness was highest in the JTD_0 group and lowest in the OTD_45 group. The rarefaction curves show that bacterial community diversity was lower in the diet treatment groups than in the control group (Supplementary Figure 6). However, soil bacterial diversity and evenness were not different between samples obtained under the different treatments in July and October (Supplementary Figure 7). Regarding fungal communities, the Chao1, Shannon, and Simpson indices were higher in the JTD_30 and OTD_30 groups than in the other groups (Supplementary Figure 8).

**TABLE 2:** Soil bacterial and fungal within habitat diversity index based on the Illumina Miseq sequencing data from July and October under different crude protein diets.

Soil samples		Microbe	Chao1	Goods_coverage	Observed_species	Pielou_e	Shannon	Simpson
July	JTD_0	Bacteria	5119.98 ± 1558.71	0.9717 ± 0.0149	4606.83 ± 1328.89	0.8731 ± 0.013	10.59 ± 0.2	0.9978 ± 0.0004
	JTD_15		4251.91 ± 155.71	0.9801 ± 0.0015	3849.57 ± 152.21	0.8797 ± 0.0065	10.48 ± 0.04	0.9978 ± 0.0004
	JTD_30		4099.02 ± 419.12	0.9802 ± 0.0028	3694.13 ± 322.66	0.8722 ± 0.0077	10.33 ± 0.2	0.9976 ± 0.0002
	JTD_45		4049.1 ± 305.11	0.9815 ± 0.005	3743.6 ± 151.92	0.8653 ± 0.0035	10.27 ± 0.07	0.9976 ± 0.0002
October	OTD_0		4090.84 ± 429.28	0.9799 ± 0.0025	3637.1 ± 343.27	0.8581 ± 0.023	10.15 ± 0.39	0.996 ± 0.0024
	OTD_15		4296.99 ± 309.15	0.9788 ± 0.0024	3842.73 ± 221.75	0.8676 ± 0.0095	10.33 ± 0.18	0.9969 ± 0.0006
	OTD_30		4074.2 ± 196.42	0.9823 ± 0.0013	3750.67 ± 213.86	0.8749 ± 0.0161	10.39 ± 0.26	0.9974 ± 0.001
	OTD_45		3861.73 ± 347.4	0.9822 ± 0.003	3515.4 ± 218.55	0.8656 ± 0.009	10.2 ± 0.18	0.9974 ± 0.0002
July	JTD_0	Fungi	102.09 ± 53.43	0.9977 ± 0.0007	91 ± 64.6149	0.43 ± 0.36	2.9967 ± 2.5825	0.59 ± 0.5
	JTD_15		102.8 ± 18.8	0.9983 ± 0.0005	98.9667 ± 17.5799	0.55 ± 0.02	3.6495 ± 0.0095	0.81 ± 0.04
	JTD_30		205.13 ± 147.3	0.9925 ± 0.0109	174.4 ± 101.9428	0.64 ± 0.17	4.5775 ± 1.0317	0.85 ± 0.12
	JTD_45		98.18 ± 8.84	0.9988 ± 0.0001	95.6333 ± 8.2851	0.68 ± 0.05	4.4956 ± 0.3902	0.89 ± 0.04
October	OTD_0		49 ± 19.4	0.9973 ± 0.0007	36.1667 ± 19.4921	0.1 ± 0.1	0.5528 ± 0.6199	0.13 ± 0.17
	OTD_15		93.93 ± 51.84	0.997 ± 0.0015	84.1 ± 52.8512	0.26 ± 0.24	1.7478 ± 1.7091	0.35 ± 0.34
	OTD_30		141.37 ± 76.47	0.9968 ± 0.003	132.3 ± 68.9519	0.59 ± 0.05	4.095 ± 0.5988	0.83 ± 0.03
	OTD_45		94.57 ± 13.75	0.9983 ± 0.0011	90.6667 ± 10.2627	0.49 ± 0.14	3.1819 ± 0.8991	0.72 ± 0.17

**FIGURE 2:**
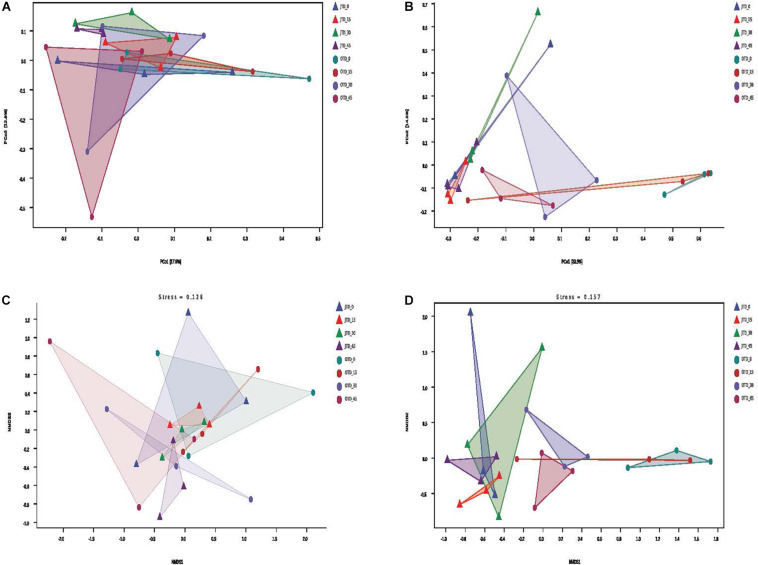
Effect of different protein content diets on soil bacterial community composition. Principal coordinate analysis (PCoA) of soil bacterial **(A)** and fungal **(B)** communities using weighted UniFrac distance metrics. Non-metric multidimensional scaling (NMDS) ordination plot of soil bacterial **(C)** and fungal **(D)** communities based on the number of OTUs detected by pyrosequencing.

Between-habitat (beta) diversity was evaluated via Principal coordinate analysis (PCoA), Non-metric Multidimensional scaling (NMDS), and cluster analysis. The PCoA based on Bray-Curtis distances accounted for 30.7% of the bacterial community variability and 47.9% of the fungal community variability (Figure 2A,B). For the fungal community, the July samples were separated from October samples in the PCoA (Figure 2B). According to the NMDS analysis, the bacterial and fungal communities had the same stress values (0.126 and 0.157, respectively); only fungal communities were different between July and October soils (Figure 2C,D).

### Soil Microbial Community Taxonomic Differences and Biomarkers

**FIGURE 3:**
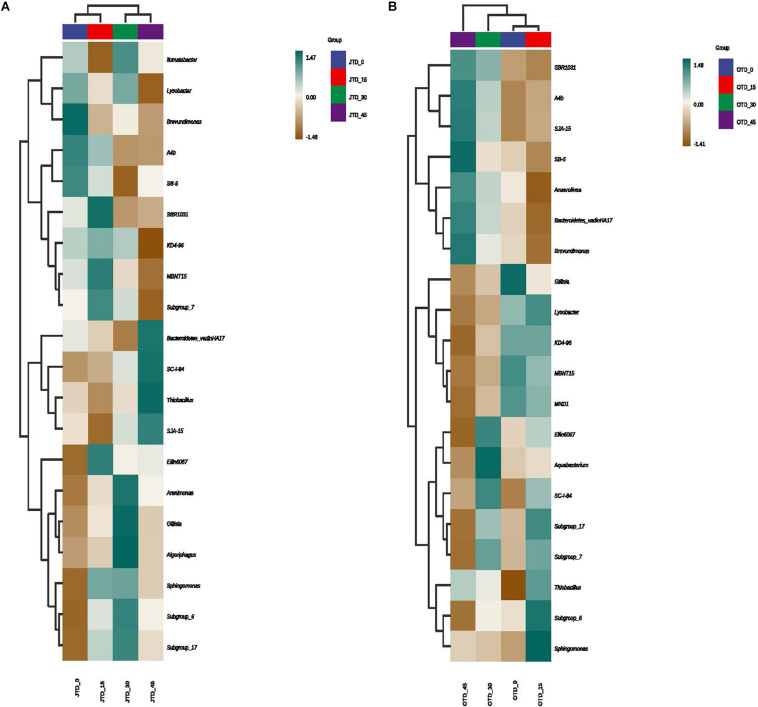
Hierarchical clustering analysis of bacterial communities under different protein content diets treatments in July **(A)** and October **(B)**.

**FIGURE 4:**
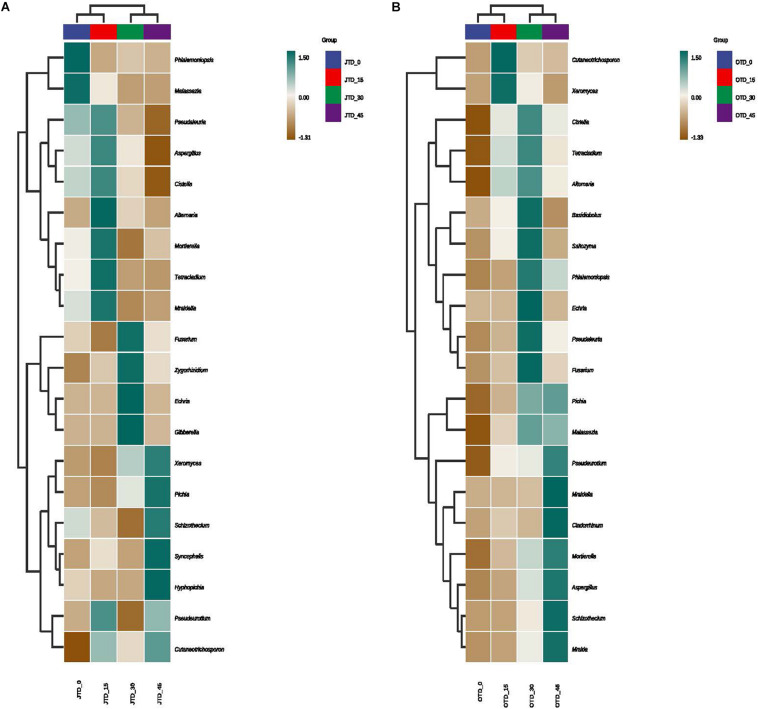
Hierarchical clustering analysis of fungal communities under different protein content diets treatments in July **(A)** and October **(B)**.

Hierarchically clustered genus-level heat-map analysis was used to identify high-dimensional biomarker taxa with significantly different abundances among the four diet treatments, comparing the samples collected in July and October. The clustering of these groups into two groups (“JTD_0 and JTD_15′’ and “JTD_30 and JTD_45′’) revealed that the bacterial and fungal taxa showed similar patterns (Figure 3). Compared to other groups, *Bacteroidetes* sp. and *SJA-15* sp. were more abundant in the JTD_45 group in both the July and October samples (Figure 3A,B). However, although *A4b*, *SB-5*, and *Brevundimonas* were less abundant in JTD_30 and JTD_45 in the July samples, they were dominant in OTD_30 and OTD_45 (Figure 3A,B). In terms of fungal genera, the relative abundance of *Phialemoniopsis*, *Pseudaleuria*, and *Echria* decreased with increased protein content in both July and October (Figure 4A,B), whereas that of *Pichia*, *Schizothecium*, and *Pseudeurotium* was higher in October than in July. In JTD_45, although *Malassezia* and *Mortierella* had lower relative abundance in July, they were dominant in October (Figure 4A,B).

## Discussion

As an important part of the paddy ecosystem, the soil microbial community enhances paddy nutrient cycling and soil health and protects the environment (Bruggen et al., 2015). However, it is highly sensitive to the effects of external agricultural chemicals such as fertilizers and pesticides (Itoh et al., 2003; Zhong and Cai, 2007; Tang et al., 2020). Rice-fish co-culture can reduce chemical application by improving land productivity and soil fertility; this is because the excreta of aquatic animals provide sufficient nutrients for the growth of soil microorganisms, which, in turn, alter the oxygen conditions of paddy surface soil (Xie et al., 2010; Wang et al., 2019). To examine these dynamics further, we compared the soil microbial community in a rice-crab co-culture paddy under different dietary protein treatments in July and October.

Our findings indicate that bacterial communities are more diverse than fungal communities. Similar results have been reported in rice-rotation systems (Ma et al., 2020; Maguire et al., 2020) and other rice co-culture systems (Li et al., 2018; Hou et al., 2021). Compared to fungi, bacteria may be more abundant in these systems because their range of metabolic and nutritional strategies enables them to adapt to more complex ecological niches and habitats. This explains why bacteria are more abundant than fungi in soils with complex environments. Although the rice-crab co-culture system can increase aquatic food supply and farmer income and help sustain rice production, the challenge of sustaining rice yield without increasing the use of fertilizer-N, pesticides, and other costly inputs is on the increase. Our results may provide referential experiences for several other countries (e.g., Egypt, India, Indonesia, Thailand, Vietnam, the Philippines, Bangladesh, Myanmar, and Malaysia) that are practicing rice co-culture.

Co-culture systems involving rice and aquatic animals cause nutrients such as carbon, nitrogen, and phosphorus to accumulate in the paddy soil, increasing its nutrient load (Wan et al., 2019; Bashir et al., 2020). However, in our study, phylum-level bacterial community composition did not change significantly with culture time, and soil bacterial diversity and evenness were not different between the July and October samples. Xu (2020) found that soil microbial biomass does not change significantly over years of co-culture (Xu, 2020). Our study site at Panjin city, which has over 30 years of co-culture history, had a higher comprehensive index of soil nutrients and decomposing bacteria (Proteobacteria and Chloroflexi) than other locations in China (Bashir et al., 2021), suggesting that Proteobacteria, Chloroflexi, and Bacteroidetes play important roles in structuring the rice-crab co-culture soil flora.

However, our beta diversity analysis revealed that the fungal community differed between the July and October samples. To our surprise, the soil mycobiota were dominated by relatively few saprotrophic fungal species instead of hundreds of bacterial taxa. Saprotrophic macrofungi such as Ascomycota and Basidiomycota are key regulators of soil nutrient cycling and decomposition. In the present study, Ascomycota was dominant in the control group, whereas Basidiomycota was the most abundant in the protein-diet treatments. Although soil macrofungi are generalists and occur in heterogeneous environments, their diversity and richness can be impacted by indiscriminate feeding during aquaculture (Crowther et al., 2012). Additionally, fungal alpha diversity indices (Chao1, Shannon, and Simpson) were higher in the JTD_30 and OTD_30 groups than in the low-protein treatment groups, indicating that saprotrophic macrofungal diversity was negatively related to dietary protein content. Although a high protein diet can effectively promote the growth performance and digestive physiology level of mitten crab, it can decrease soil pH from the rice-crab coculture system and negatively impact soil bacteria community diversity. Moreover, formulated feed does not make up a larger portion of the diet of *E. sinensis*, and excess formulated feed added to the rice-crab system sinks to the bottom of field, which may lead to water pollution (Guo et al., 2015). Therefore, a lower dietary protein level such as ca. 15% is appropriate for the rice-crab co-culture system both environmentally and economically.

In both July and October samples, the high-protein groups (JTD_30, JTD_45, OTD_30, and OTD_45) had greater relative abundances of Bacteroidetes and Thiobacillus than the control and low-protein groups (JTD_0, JTD_15, OTD_0, and OTD_15). We hypothesize that high-protein feeding might gradually reduce soil pH while promoting organic matter, nitrate nitrogen, phosphate, and other nutrient accumulation. This hypothesis is supported by previous findings that the relative abundance of Bacteroidetes and Thiobacillus increases during biogas irrigation (Yuge et al., 2014). The occurrence of these bacteria in the rice soil microbiota may reflect its physiological and biochemical conditions, such as its high organic matter, protein content, and pH. Therefore, soil pH may play a critical role in driving the topological structure of the co-occurrence network for soil microbiota, and the network was the most stable at neutral pH (Chen et al., 2021).

*Brevundimonas*, as a plant growth-promoting rhizobacterial genus, enhances the yield and growth of rice (Singh et al., 2016), potato (Naqqash et al., 2020), and wheat (Anuj et al., 2012), thereby allowing reduced application of chemical fertilizers and causing minimal environmental impact. We found that high-protein diets (JTD_30 and JTD_45) significantly reduced the relative abundance of *Brevundimonas* in both the July and October samples.

## Conclusion

In conclusion, we evaluated how the soil microbial community of a rice-crab co-culture system responded to different dietary protein levels using partial 16S/18S rRNA gene analysis. A number of dominant and rare bacterial phyla (groups) were detected. Our results indicated that bacterial community structure was not affected by diet treatment, while in fungi, significant community structure change was evident. Moreover, high-protein diets reduced Chao 1 index and the Observed_species index in both July and October. Low-protein diet soils displayed greater bacterial diversity than high-protein diets soils, which reveal that a dietary protein level of ca. 15% is appropriate for this rice-crab co-culture system. Owing to the limited sampling and restricted sampling area (Panjin City, northern China), these conclusions cannot be generalized to other locations. However, our findings represent an important step toward understanding how diet alters soil microbial communities in rice–crab co-culture systems. In the future, we will conduct a more comprehensive study of the interaction between feeding patterns and soil microbial communities in rice-crab co-culture systems to formulate more accurate recommendations for this co-culture model.

## Data Availability Statement

The data presented in the study are deposited in the NCBI Sequence Read Archive (SRA) database and Biosample accession number was SAMN17393294.

## Author Contributions

YL and XL designed the experiments, analyzed the data, contributed reagents, materials, and analysis tools, and wrote the manuscript. YY and LL generated biological samples. YY, LL, and QH performed the experiments. YL and LL performed statistical analysis. All authors read and approved the final manuscript.

## Conflict of Interest

The authors declare that the research was conducted in the absence of any commercial or financial relationships that could be construed as a potential conflict of interest.

## Footnotes

1 https://docs.qiime2.org/2019.4/tutorials/

## Supplementary Material

Supplementary Figure 1 Length distribution of ASV/OUT sequences of bacterial group under different protein content diets treatments in July and October.

Supplementary Figure 2 Length distribution of ASV/OUT sequences of fugal group under different protein content diets treatments in July and October.

Supplementary Figure 3 Annotation of ASV/OUT sequences of bacterial group under different protein content diets treatments in July and October.

Supplementary Figure 4 Annotation of ASV/OUT sequences of fugal group under different protein content diets treatments in July and October.

Supplementary Figure 5 Top ten bacterial **(A)** and fungal **(B)** in phyla level under different protein content diets treatments in July and October.

Supplementary Figure 6 Rarefaction curves of bacterial group under different protein content diets treatments in July and October.

Supplementary Figure 7 Chao1 and Simpson indices of bacterial group under different protein content diets treatments in July and October.

Supplementary Figure 8 Chao1 and Simpson indices of fungal group under different protein content diets treatments in July and October.

## References

[B1] Anuj R. Baljeet S. Lata N. Radha R. Shivay Y. S. (2012). Enhancing micronutrient uptake and yield of wheat through bacterial PGPR consortia(Plant nutrition). *Soil Sci. Plant Nutr.* 58 573–582. 10.1080/00380768.2012.716750

[B2] Bashir M. A. Liu J. Geng Y. Wang H. Pan J. Zhang D. (2020). Co-culture of rice and aquatic animals: an integrated system to achieve production and environmental sustainability. *J. Clean. Prod.* 249:119310. 10.1016/j.jclepro.2019.119310

[B3] Bashir M. A. Wang H. Pan J. Khoshnevisan B. Sun W. Zhai L. (2021). Variations in soil nutrient dynamics and their composition in rice under integrated rice-crab co-culture system. *J. Clean. Prod.* 281:125222. 10.1016/j.jclepro.2020.125222

[B4] Bruggen A.H.C.v Sharma K. Kaku E. Karfopoulos S. Zelenev V. V. Blok W. J. (2015). Soil health indicators and *Fusarium* wilt suppression in organically and conventionally managed greenhouse soils. *Appl. Soil Ecol.* 86 192–201. 10.1016/j.apsoil.2014.10.014

[B5] Chen B. Jiao S. Luo S. Ma B. Qi W. Cao C. (2021). High soil pH enhances the network interactions among bacterial and archaeal microbiota in alpine grasslands of the Tibetan Plateau. *Environ. Microbiol.* 23 464–477. 33215802 10.1111/1462-2920.15333

[B6] Crowther T. W. Boddy L. Jones T. H. (2012). Functional and ecological consequences of saprotrophic fungus-grazer interactions. *ISME J.* 6 1992–2001. 10.1038/ismej.2012.53 22717883 PMC3475375

[B7] Guo K. Zhao W. Li W. K. Zhao Y. S. Zhang P. Zhang C. (2015). Food web structure and trophic levels in polyculture rice-crab fields. *Chin. J. Oceanol. Limnol.* 33 735–740. 10.1007/s00343-015-4205-8

[B8] Hou J. Styles D. Cao Y. Ye X. (2021). The sustainability of rice-crayfish coculture systems: a mini review of evidence from Jianghan Plain in China. *J. Sci. Food Agric.* 101 3843–3853. 10.1002/jsfa.11019 33336495

[B9] Hu L. Guo L. Zhao L. Shi X. Ren W. Zhang J. (2020). Productivity and the complementary use of nitrogen in the coupled rice-crab system. *Agric. Syst.* 178:102742. 10.1016/j.agsy.2019.102742

[B10] Hu L. Zhang J. Ren W. Guo L. Cheng Y. Li J. (2016). Can the co-cultivation of rice and fish help sustain rice production? *Sci. Rep.* 6:28728. 10.1038/srep28728 27349875 PMC4923892

[B11] Itoh K. Ikushima T. Suyama K. Yamamoto H. (2003). Evaluation of pesticide effects on microbial communities in a paddy soil comparing with that caused by soil flooding. *J. Pestic. Sci.* 28 51–54. 10.1584/jpestics.28.51 27476087

[B12] Lawler S. P. (2001). Rice fields as temporary wetlands: a review. *Israel J. Zool.* 47 513–528. 10.1092/X7K3-9JG8-MH2J-XGX1 26920470

[B13] Li P. Ye S. Liu H. Pan A. Ming F. Tang X. (2018). Cultivation of drought-tolerant and insect-resistant rice affects soil bacterial, but not fungal, abundances and community structures. *Front. Microbiol.* 9:1390. 10.3389/fmicb.2018.01390 30008701 PMC6033987

[B14] Ma X. Du M. Liu P. Tang Y. Liu Z. (2020). Alternation of soil bacterial and fungal communities by tomato–rice rotation in Hainan Island in Southeast of China. *Arch. Microbiol.* 203 913–925. 10.1007/s00203-020-02086-5 33078269

[B15] Maguire V. G. Bordenave C. D. Nieva A. S. Llames M. E. Colavolpe M. B. Gárriz A. (2020). Soil bacterial and fungal community structure of a rice monoculture and rice-pasture rotation systems. *Appl. Soil Ecol.* 151:103535. 10.1016/j.apsoil.2020.103535

[B16] Naqqash T. Imran A. Hameed S. Shahid M. Ejaz S. (2020). First report of diazotrophic *Brevundimonas* spp. as growth enhancer and root colonizer of potato. *Sci. Rep.* 10:12893. 10.1038/s41598-020-69782-6 32732939 PMC7393102

[B17] Sha Z. Chu Q. Zhao Z. Yue Y. Lu L. Yuan J. (2017). Variations in nutrient and trace element composition of rice in an organic rice-frog coculture system. *Sci. Rep.* 7 1–10. 10.1038/s41598-017-15658-1 29146988 PMC5691045

[B18] Singh N. Marwa N. Mishra S. K. Mishra J. Verma P. C. Rathaur S. (2016). *Brevundimonas diminuta* mediated alleviation of arsenic toxicity and plant growth promotion in Oryza sativa L. *Ecotoxicology and Environ. Saf.* 125 25–34. 10.1016/j.ecoenv.2015.11.020 26650422

[B19] Song C. Zhang J. Hu G. Meng S. Fan L. Zheng Y. (2019). Risk assessment of chlorantraniliprole pesticide use in rice-crab coculture systems in the basin of the lower reaches of the Yangtze River in China. *Chemosphere* 230 440–448. 10.1016/j.chemosphere.2019.05.097 31121508

[B20] Stuart A. M. Devkota K. P. Sato T. Pame A. R. P. Balingbing C. Phung N. T. M. (2018). On-farm assessment of different rice crop management practices in the Mekong Delta, Vietnam, using sustainability performance indicators. *Field Crops Res.* 229 103–114. 10.1016/j.fcr.2018.10.001

[B21] Tang X. Li L. Wu C. Khan M. I. Manzoor M. Zou L. (2020). The response of arsenic bioavailability and microbial community in paddy soil with the application of sulfur fertilizers. *Environ. Pollut.* 264:114679. 10.1016/j.envpol.2020.114679 32380397

[B22] Wan N.-F. Li S.-X. Li T. Cavalieri A. Weiner J. Zheng X.-Q. (2019). Ecological intensification of rice production through rice-fish co-culture. *J. Clean. Prod.* 234 1002–1012. 10.1016/j.jclepro.2019.06.238

[B23] Wang X. Xiao B. Hu K. (2019). Rice-crab coculture to sustain cleaner food production in Liaohe River Basin, China: an economic and environmental assessment. *J. Clean. Prod.* 208 188–198. 10.1016/j.jclepro.2018.10.092

[B24] Xie J. Hu L. Tang J. Wu X. Li N. Yuan Y. (2011). Ecological mechanisms underlying the sustainability of the agricultural heritage rice–fish coculture system. *Proc. Natl. Acad. Sci.* 108 1381–1387. 10.1073/pnas.1111043108 22084110 PMC3250190

[B25] Xie J. Wu X. Tang J. Zhang J. Chen X. (2010). Chemical fertilizer reduction and soil fertility maintenance in rice-fish coculture system. *Front. Agric. China* 4:422–429. 10.1007/s11703-010-1049-z

[B26] Xu Y. (2020). *A Preliminary Study on the Effects of Procambarus Clarkii Aquaculture on Paddy Field Ecosystem*. Master dissertation. Dalian: Dalian Ocean University.

[B27] Yao Z. Zheng X. Liu C. Lin S. Zuo Q. Butterbach-Bahl K. (2017). Improving rice production sustainability by reducing water demand and greenhouse gas emissions with biodegradable films. *Sci. Rep.* 7 1–10. 10.1038/srep39855 28054647 PMC5214061

[B28] Yuge K. Maeda H. Tanaka M. Anan M. Shinogi Y. (2014). Spatial-uniform application method of methane fermentation digested slurry with irrigation water in the rice paddy field. *Paddy Water Environ.* 12 335–342. 10.1007/s10333-013-0382-2

[B29] Zhong W. H. Cai Z. C. (2007). Long-term effects of inorganic fertilizers on microbial biomass and community functional diversity in a paddy soil derived from quaternary red clay. *Appl. Soil Ecol.* 36 84–91. 10.1016/j.apsoil.2006.12.001

