# Supplementary figures and images for: Impact of Dietary Protein Content on Soil Bacterial and Fungal Communities in a Rice–Crab Co-culture System
Source: Front Microbiol. 2021 Jun 21;12:696427. doi: 10.3389/fmicb.2021.696427 (PMC8256891; doi:10.3389/fmicb.2021.696427)

Length Distribution

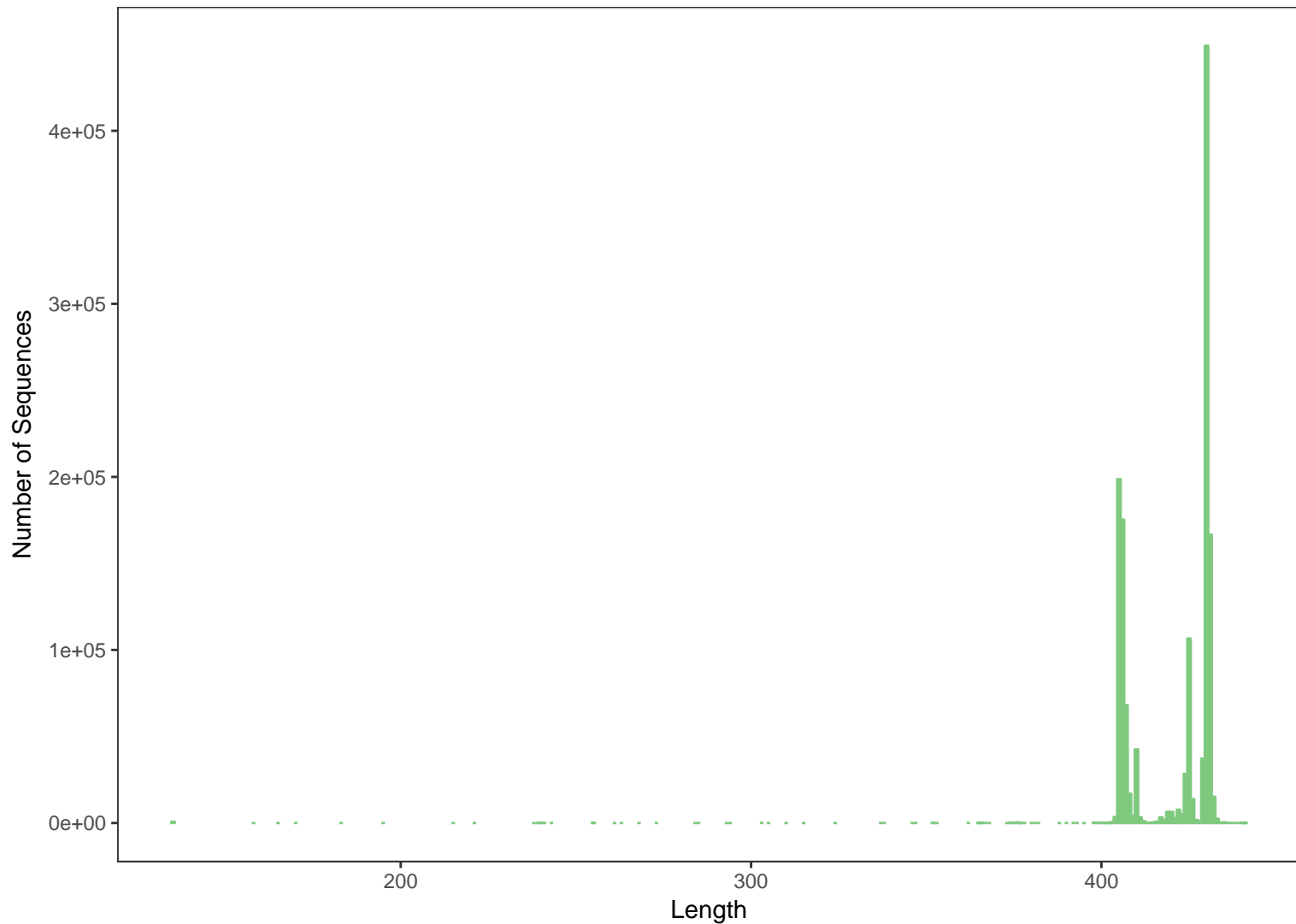

Supplement: Supplementary Figure 1 — Length distribution of ASV/OUT sequences of bacterial group under different protein content diets treatments in July and October. [file Data_Sheet_1.PDF]

## Length Distribution

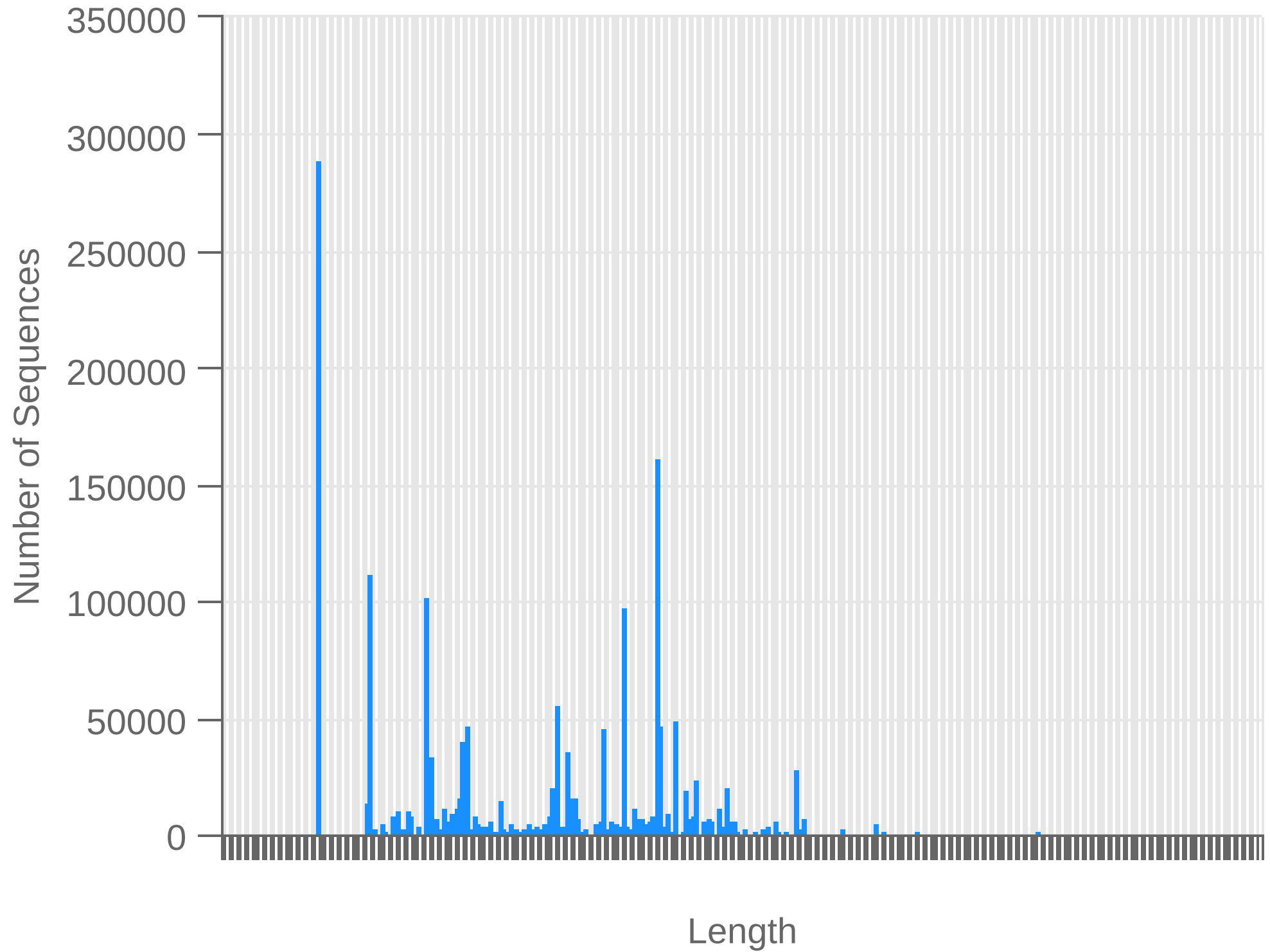

Supplement: Supplementary Figure 2 — Length distribution of ASV/OUT sequences of fugal group under different protein content diets treatments in July and October. [file Data_Sheet_2.PDF]

The Number of ASVs/OTUs

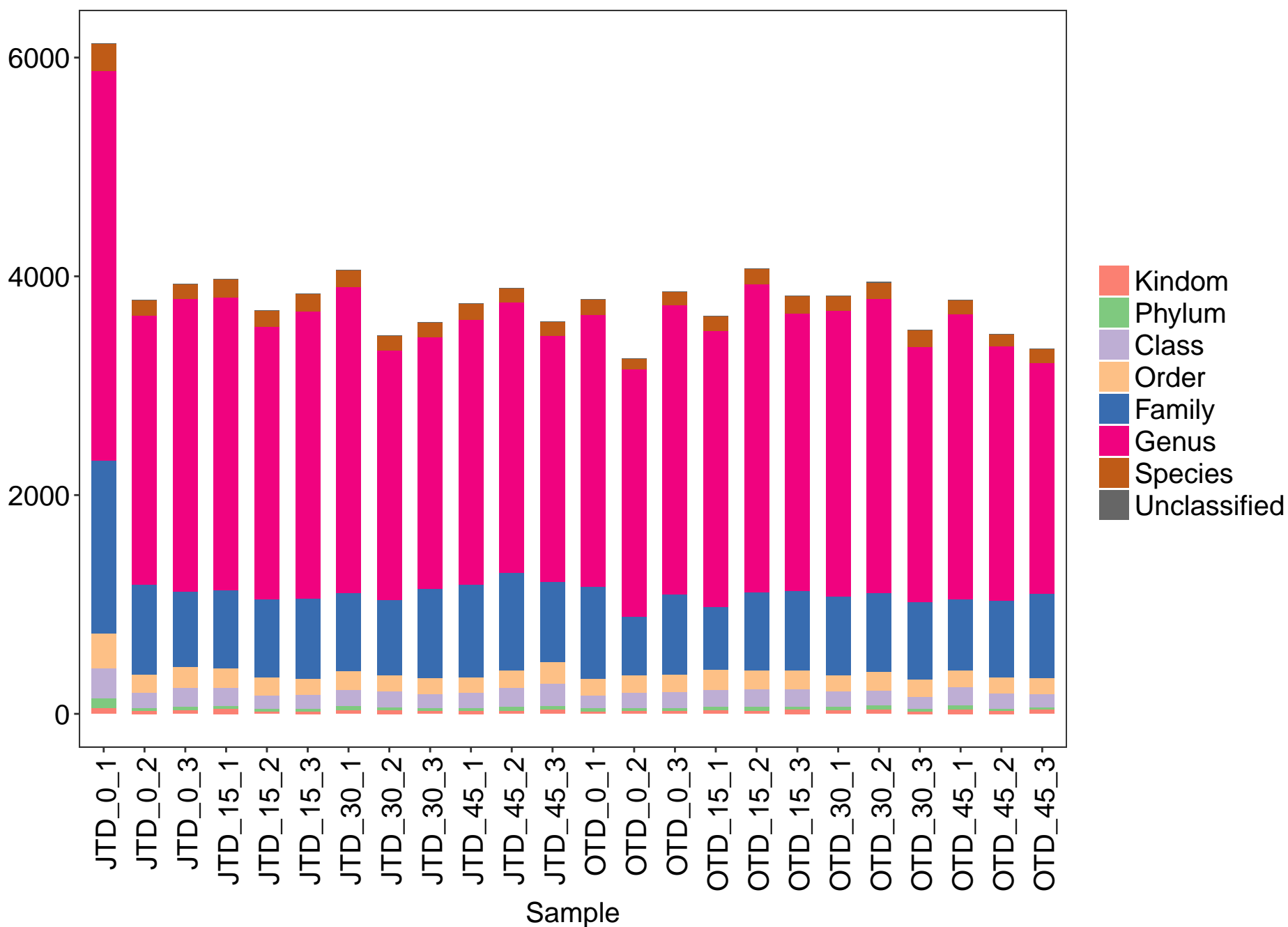

Supplement: Supplementary Figure 3 — Annotation of ASV/OUT sequences of bacterial group under different protein content diets treatments in July and October. [file Data_Sheet_3.PDF]

The Number of ASVs/OTUs

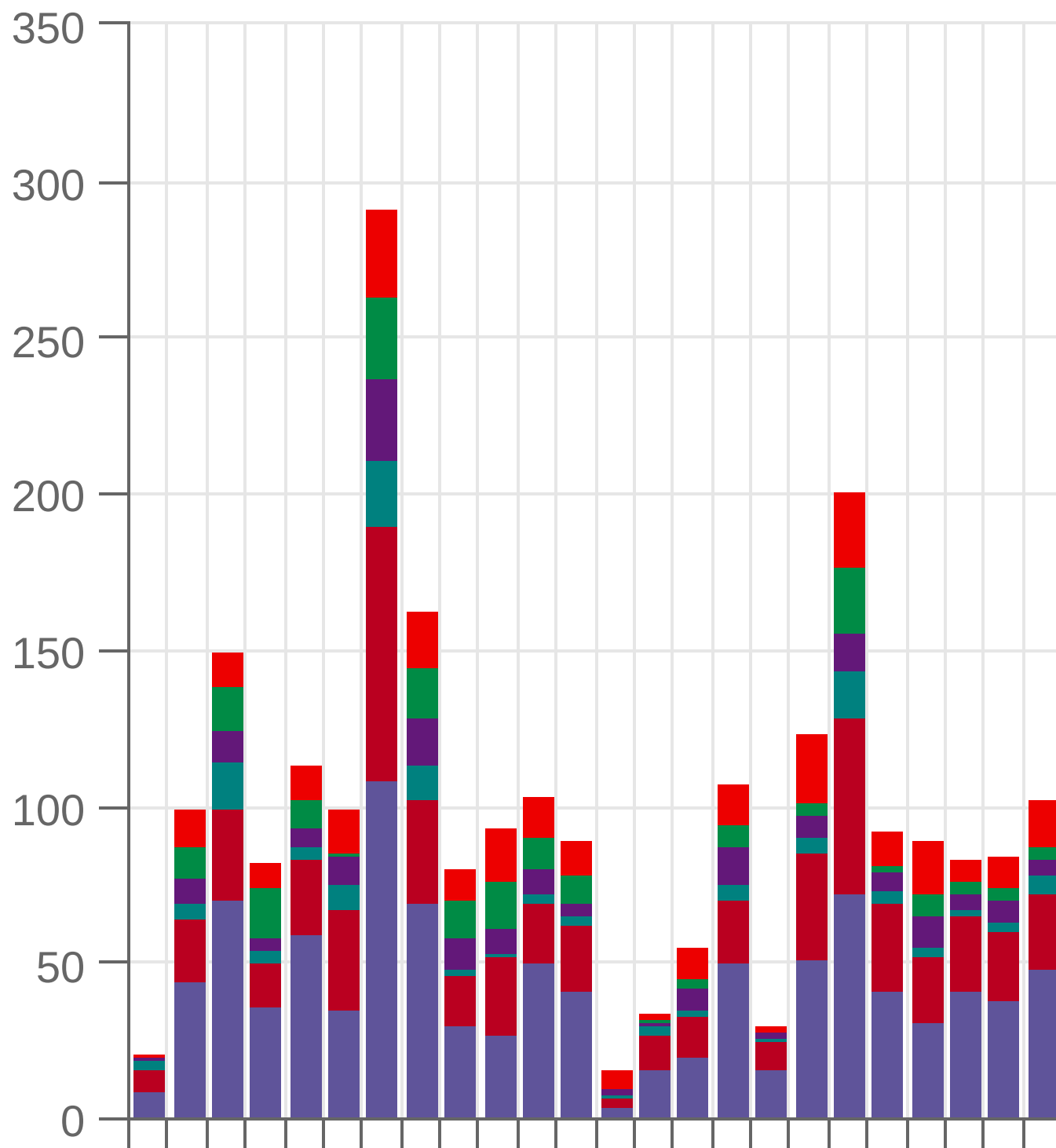

- unclassified
- species
- genus
- family
- order
- class
- phylum
- domain

Sample

Supplement: Supplementary Figure 4 — Annotation of ASV/OUT sequences of fugal group under different protein content diets treatments in July and October. [file Data_Sheet_4.PDF]

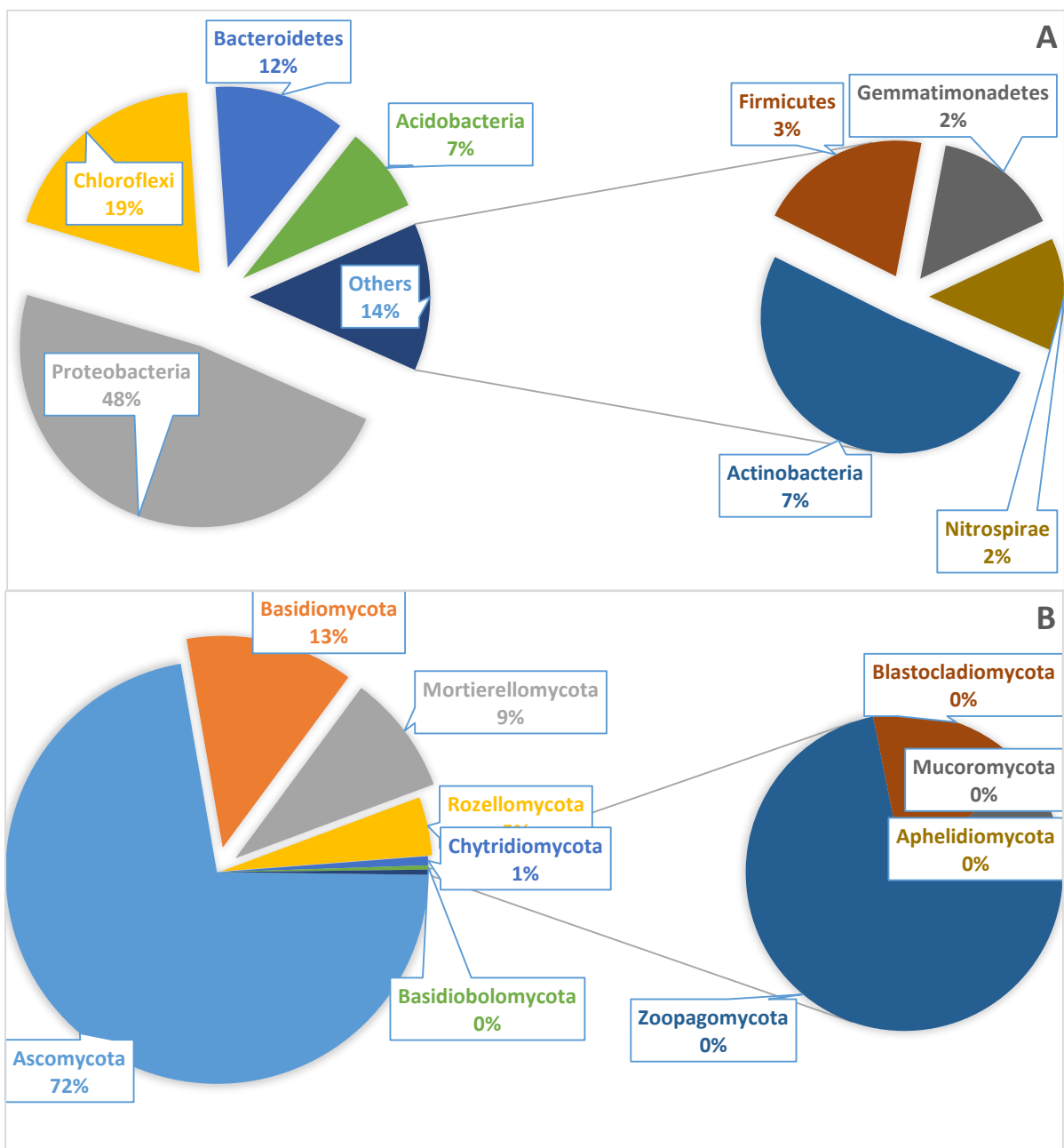

Supplement: Supplementary Figure 5 — Top ten bacterial (A) and fungal (B) in phyla level under different protein content diets treatments in July and October. [file Data_Sheet_5.PDF]

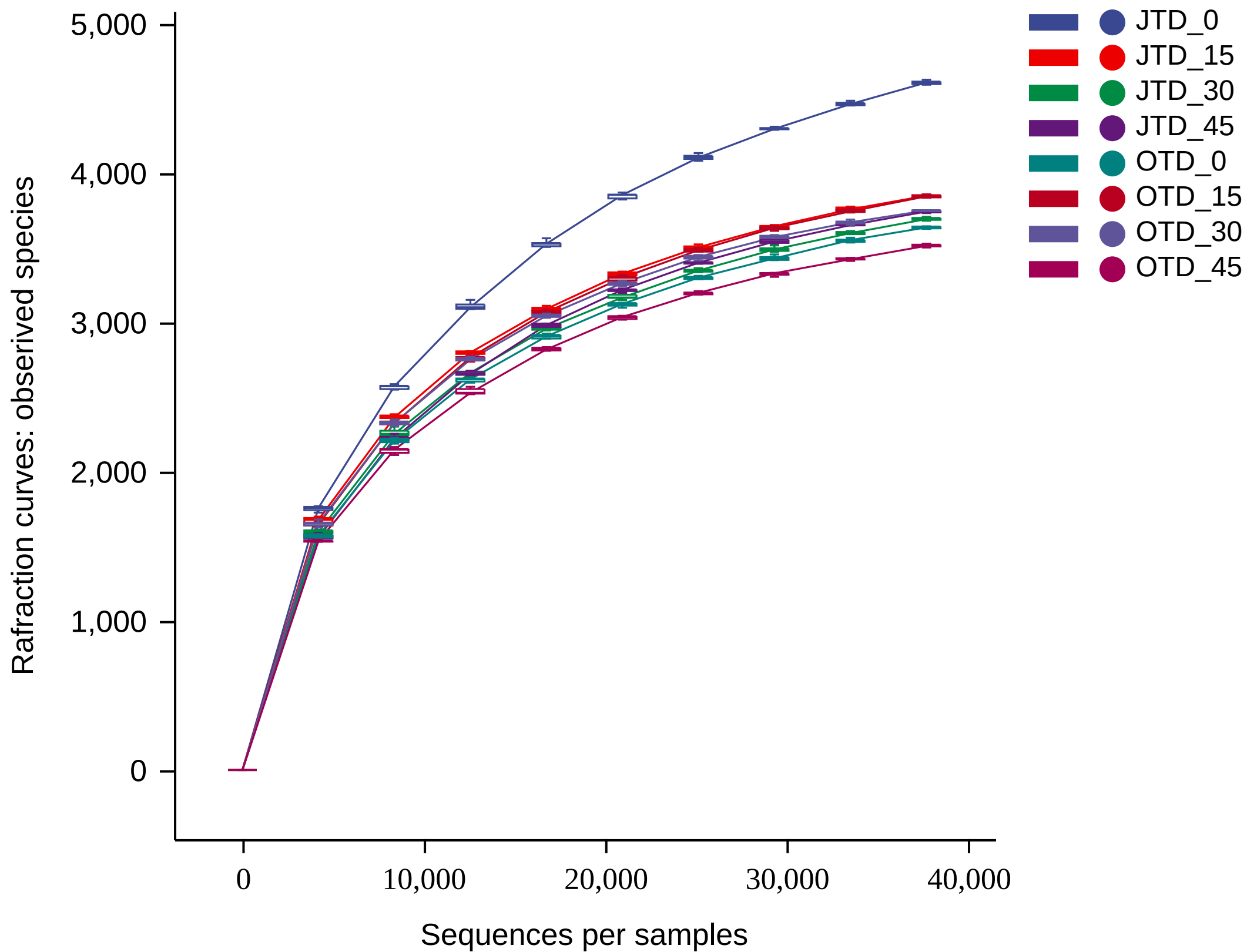

Supplement: Supplementary Figure 6 — Rarefaction curves of bacterial group under different protein content diets treatments in July and October. [file Data_Sheet_6.PDF]

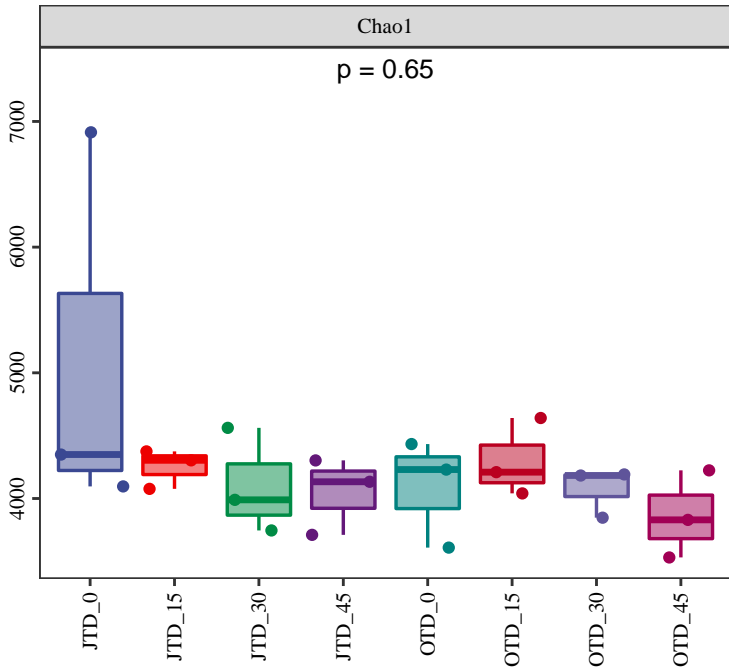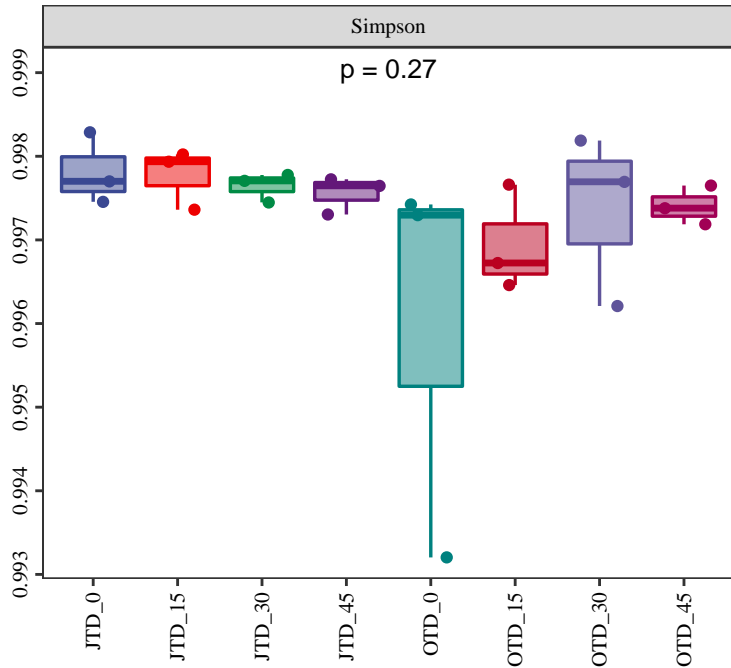

Bacterial

JTD\_0

JTD\_15

JTD\_30

JTD\_45

OTD\_0

OTD\_15

OTD\_30

OTD\_45

Supplement: Supplementary Figure 7 — Chao1 and Simpson indices of bacterial group under different protein content diets treatments in July and October. [file Data_Sheet_7.PDF]

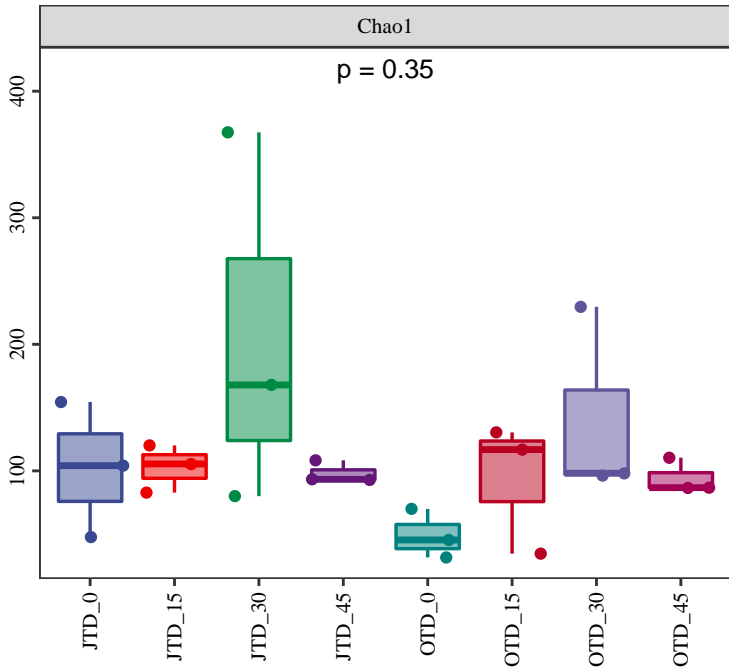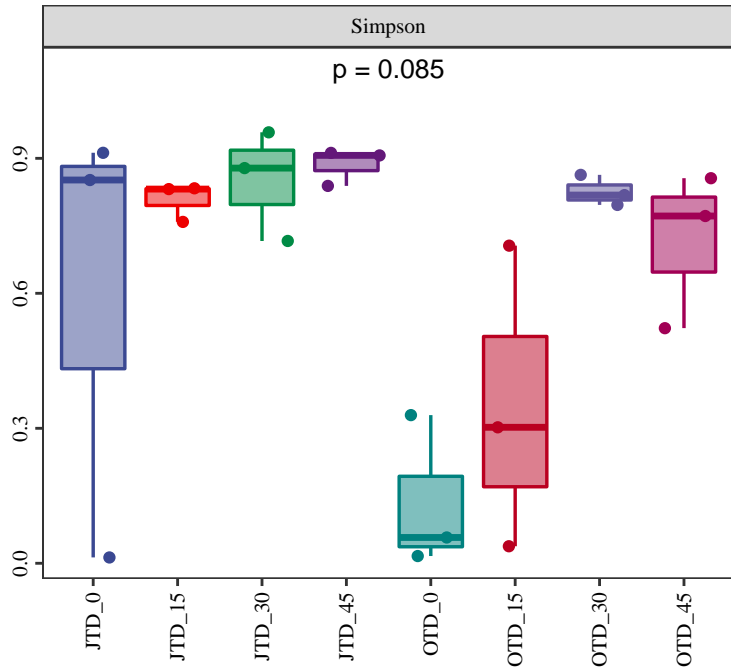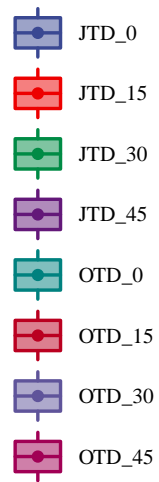

Supplement: Supplementary Figure 8 — Chao1 and Simpson indices of fungal group under different protein content diets treatments in July and October. [file Data_Sheet_8.PDF]
